# Associations Between Pornography Consumption, Sexual Flexibility, and Sexual Functioning Among Austrian Adults

**DOI:** 10.1007/s10508-021-02201-7

**Published:** 2022-01-04

**Authors:** Nikola Komlenac, Margarethe Hochleitner

**Affiliations:** Gender Medicine Unit, Medical University of Innsbruck, Fritz-Pregl Strasse 3, 6020 Innsbruck, Austria

**Keywords:** Pornography consumption, Sexual functioning, Sexual flexibility, Young adults, Acquisition, activation, application model (_3_AM), Austria

## Abstract

To date, only a few studies have examined the associations between pornography consumption and sexual functioning. The Acquisition, Activation, Application Model (_3_AM) indicates that the frequency of pornography consumption and the perceived realism of pornography may influence whether sexual scripts are acquired from viewed pornography. Having sexual scripts that are alternative to their preferred sexual behaviors may help people switch to alternative sexual behavior when sexual problems arise. The current study analyzed whether frequent pornography consumption was associated with greater sexual flexibility and greater sexual functioning. Additionally, the perceived realism of pornography consumption was tested as a moderator of those associations. At an Austrian medical university, an online cross-sectional questionnaire study was conducted among 644 medical students (54% women and 46% men; *M*_age_ = 24.1 years, SD = 3.8). The participants were asked about their pornography consumption, partnered sexual activity, sexual flexibility, perceived realism of pornography, and sexual functioning. Manifest path analyses revealed direct and indirect associations between frequent pornography consumption and greater sexual functioning through greater sexual flexibility in women but not in men. Perceived realism did not moderate those associations. In conclusion, our study was in line with previous studies that found no significant associations between men’s pornography consumption and sexual functioning in men. However, some women may expand their sexual scripts and learn new sexual behaviors from pornography consumption, which may help with their sexual functioning.

## Introduction

The majority of adults in the USA (91.9% of women and 98.9% of men) have consumed pornography at least once in their lifetime (Solano et al., 2020). Similarly high numbers for pornography consumption have been reported by European studies (Donevan & Mattebo, 2017; Häggström-Nordin et al., 2005; Miller et al., 2019). On average, men report consuming pornography once or more times a week (Landripet & Štulhofer, 2015; Miller et al., 2019; Sun et al., 2016). Women consume pornography on average less frequently than men do. Namely, women consume pornography on average several times a month (Grubbs et al., 2021; Kohut et al., 2017).

Most people who consume pornography either do not perceive any consequences of their pornography consumption (Malki et al., 2021) or they self-perceive positive consequences of pornography consumption, such as receiving information on how to expand their repertoire of sexual behavior (Kohut et al., 2017). However, in contrast to consumers’ self-perceived consequences of their pornography consumption, many studies report links between pornography consumption and poor sexual health (Leonhardt & Willoughby, 2019; Miller et al., 2019; Wright et al., 2017, 2019). Studies on the associations between pornography consumption and one aspect of sexual health, namely, sexual functioning, are scarce, and the existing results are inconclusive (Dwulit & Rzymski, 2019; Grubbs & Gola, 2019).

The current study expands the current literature by examining the associations between pornography consumption and sexual functioning in an Austrian sample of women and men. Based on consumers’ self-perceived effects of their pornography consumption (Daneback et al., 2009; Hald & Malamuth, 2008; Weinberg et al., 2010), in the current study, it was hypothesized that pornography consumption would be associated with greater sexual flexibility (Gauvin & Pukall, 2018) and that, in turn, greater sexual flexibility would be linked with greater sexual functioning.

### Pornography

Pornography can be defined as “material [e.g., pictures, films, videos or text] deemed sexual, given the context, that has the primary intention of sexually arousing the consumer, and is produced and distributed with the consent of all persons involved” (McDonald & Kirkman, 2019, p. 163). Central in the definition of pornography is the consent of all persons involved. Therefore, materials that were produced or distributed without the consent of at least one person involved (e.g., “revenge porn,” “child pornography”) were excluded from this definition (McDonald & Kirkman, 2019).

Currently, pornographic material contains depictions of a sheer variety of sexual behaviors and sexual activities (Klaassen & Peter, 2015; McKee, 2005). Most commonly, two actors are featured, and the depicted behaviors include genital stimulation, oral stimulation, or vaginal intercourse (Gorman et al., 2010; Vannier et al., 2014).

However, many pornographic materials can be criticized because one partner is often portrayed as an object of degradation or objectification by the other(s). In most heterosexual portrayals of sexual activity, women experience degradation or objectification (McKee, 2005). Additionally, domination and exploitation can be common themes in pornographic materials (Gorman et al., 2010), whereby women are more frequently portrayed as submissive, whereas men are more frequently portrayed as dominant (Klaassen & Peter, 2015). Such portrayals of domination and submissiveness can also include aggression (Shor & Seida, 2019). Furthermore, male pleasure is often emphasized, whereas women are often portrayed as having the sole purpose of sexually gratifying their partner(s) (Gorman et al., 2010).

The high availability, the frequent use of pornographic material and the portrayal of sexual activity in pornographic material that includes degradation, objectification, domination, exploitation, or aggression all lead to the premise that pornographic materials may be linked to people exhibiting partnered sexual activities that are similar to those depicted in pornographic material (Braithwaite et al., 2015; Herbenick et al., 2020). For example, a German study revealed an association between men’s frequency of pornography consumption and their engagement in dominant sexual activities (e.g., choking a partner, ejaculating on a partner’s face, name calling) (Wright et al., 2015). Another German study revealed a correlation between women’s frequency of pornography consumption and their desire for or actual submissive sexual behaviors (e.g., being spanked by a partner, being slapped by a partner in the face, being choked by a partner) (Sun et al., 2017).

### Acquisition, Activation, Application Model

One model that might explain such associations between pornography consumption and consumers’ sexual behavior is the Acquisition, Activation, Application Model (_3_AM) (Wright, 2011). According to the _3_AM, people may acquire new mental representations or sexual scripts of how partnered sexual activities should be from pornography consumption. Such sexual scripts are cognitive knowledge structures in one’s memory that contain information about the partner(s), the sequence of and type of certain sexual behaviors, and the consequences of certain sexual behaviors. According to the model, a number of content variables may influence whether new sexual scripts are acquired from pornography consumption, such as frequency of consumption or perceived realism of pornography. Therefore, frequent pornography consumption and the perception of pornography being realistic may increase the likelihood of pornographic content being learned and acquired as sexual scripts. The activation of a sexual script occurs when persons perceive certain cues that help them easily retrieve a certain sexual script from memory. Such cues may be perceived during partnered sexual activity and trigger sexual scripts to be activated. Finally, a person may try to apply an activated sexual script to a certain sexual situation, and the person may try to behave and expect the other(s) to behave according to the particular sexual script (Wright, 2011; Wright & Bae, 2016).

Because of the sheer variability of sexual behaviors portrayed in pornography, many sexual scripts learned from pornography may not be applicable to partnered sexual activity. Failing to apply a certain sexual script to a partnered sexual activity may be frustrating or dissatisfying. Furthermore, some people may start to prefer sexual behaviors that are depicted in pornography (Bridges et al., 2016), or they may prefer masturbation while consuming pornography to partnered sexual activity (Berger et al., 2019). Thus, it is often reported that pornography consumption is linked to relationship dissatisfaction and problems (Miller et al., 2019), sexual dissatisfaction (Wright et al., 2019), or problems with sexual functioning.

### Sexual Functioning

However, studies about the associations between pornography consumption and sexual functioning, which is defined as “a person's ability to respond sexually or to experience sexual pleasure” (American Psychiatric Association, 2013, p. 423), are inconclusive. Few studies deal with women’s pornography consumption and the link to their sexual functioning. Of those, one study with a small sample (*n* = 48) of U.S. women reported that there were no associations between pornography consumption and women’s sexual functioning (Berger et al., 2019). Larger studies have revealed links between frequent pornography consumption and more difficulties with reaching orgasm in women (McNabney et al., 2020; Wright et al., 2021).

Additionally, in men, links between frequent pornography consumption and worse sexual functioning have been reported in the past. For instance, an association between increased pornography consumption and worse erectile functioning has been revealed (Grubbs & Gola, 2019; Malki et al., 2021). Furthermore, men who prefer masturbation while consuming pornography have been found to be more likely to be affected by sexual problems than are men who prefer partnered sexual activity. In contrast, a study among 1211 sexually active heterosexually identified Croatian men could not replicate such a finding. In that study, no associations between pornography consumption and sexual problems were observed (Landripet & Štulhofer, 2015). Similarly, a U.S. study among 127 heterosexually identified men reported no association between pornography consumption and erectile problems (Prause & Pfaus, 2015). In addition, there are also studies that report links between frequent pornography consumption and fewer sexual problems (Bőthe et al., 2021).

### Sexual Flexibility

Supporting findings showing that pornography consumption may be associated with positive experiences during partnered sexual activity come from studies that report consumers’ self-perceived effects of their pornography consumption (Daneback et al., 2009; Hald & Malamuth, 2008; Weinberg et al., 2010). Many people use pornographic material to enhance their sexuality (Daneback et al., 2009; Rothman et al., 2021). Pornography can be used as a means of exploring one’s own sexuality. People report that pornography helps them determine and understand their sexual interests, their preferences or their sexual identities (McCormack & Wignall, 2017). During pornography consumption, they can get to know their bodily reactions to sexual stimulation. Sexual behavior depicted in pornography is also used by some people to learn and get to know new sexual activities and new ideas that can be applied to partnered sexual activities with positive consequences (Attwood et al., 2018). People report that some pornographic material has helped them expand their repertoire of sexual behavior and that it has inspired them to undertake sexual experimentation during partnered sexual activity (Kohut et al., 2017). After consuming pornography, some people realized that they found more sexual activities appealing and that they were also encouraged to try out new sexual activities. Learning about oral-genital sexual behaviors was most frequently cited in this regard (Weinberg et al., 2010).

The _3_AM informs that sexual scripts are more likely acquired when viewed pornographic content is perceived as being realistic or when the content is similar to other pre-existing sexual scripts that the person acquired through other means (e.g., personal experience). Such scripts that seem to be similar to partnered sexual activity may, when activated during a partnered sexual activity, also be applicable to partnered sexual activity and result in positive sexual experiences (Wright, 2011; Wright & Bae, 2016).

Thus, having alternative sexual scripts to the preferred sexual activity or being open to trying out new sexual activities may help people switch to alternative (rewarding) sexual behavior when sexual problems arise (e.g., the preferred method of stimulation does not result in the expected sexual arousal). The current study analyzed whether such so-called sexual flexibility (Gauvin & Pukall, 2018) would mediate the association between sexual functioning and pornography consumption. Namely, we expected a link between increased pornography consumption and increased sexual flexibility. In turn, we expected an association between greater sexual flexibility and better sexual functioning.

### Aim of the Study

In summary, studies about the associations between pornography consumption and sexual functioning are inconclusive (Grubbs & Gola, 2019). Furthermore, there is a research gap regarding associations between pornography consumption and women’s sexual functioning. Pornography consumption may be linked to poorer sexual functioning during partnered sexual activity because pornography consumers may try to apply sexual scripts to partnered sexual activity that are not rewarding or do not lead to the same positive consequences as those portrayed in pornography. However, there may also be an association between pornography consumption and greater sexual functioning. Pornography consumption may be linked to greater sexual flexibility on the part of pornography consumers. Studies on pornography consumers’ self-perceived effects of their pornography consumption have indicated that pornography consumers learn a variety of sexual behaviors that may positively influence their sexual flexibility (Kohut et al., 2017). In turn, greater sexual flexibility may be associated with greater sexual functioning.

The current study uses a sample of young Austrian adults to address the following hypotheses:

#### H1

Frequent pornography consumption is associated with greater sexual functioning in women and men.

#### H2

This association is mediated by sexual flexibility.

#### H3

This link between pornography consumption, sexual flexibility and sexual functioning is stronger for people who perceive pornography as being realistic than for people who do not perceive such content to be realistic. Thus, perceived realism moderates the associations between pornography consumption, sexual flexibility and sexual functioning.

## Method

### Participants

Of the 644 respondents, 350 (54%) were women, and 294 (46%) were men. The male participants (*M* = 24.5, SD = 3.8) were older than the female participants (*M* = 23.6, SD = 3.8; *t*(642) = 3.0, *p* = .003, *r* = .12). Most of the participants reported holding Austrian or German nationality. Some reported holding Italian nationality or indicated “other nationality” (Table 1). The majority of the sample identified as heterosexual. More female than male participants identified with a nonheterosexual sexual orientation. The majority of the sample reported being in a relationship at the time of the study (Table 1).

**Table 1:** Sociodemographic characteristics of participants (*N* = 644)

Characteristic	Full sample		Women		Men		df	*χ*^2^
	*n*	%	*n*	%	*n*	%		
*Nationality*							3	3.4
Austrian	385	59.8	211	60.3	174	59.2		
German	147	22.8	72	20.6	75	25.5		
Italian	90	14.0	55	15.7	35	11.9		
Other	22	3.4	12	3.4	10	3.4		
*Sexual orientation*							2	10.9*
Heterosexual	565	87.9	302	86.3	264	89.8		
Gay-identified/lesbian-identified	19	3.0	6	1.7	13	4.4		
Bisexual	59	9.2	42	12.0	17	5.8		
*Relationship*							1	10.1*
Single	238	37.0	110	31.4	128	43.5		
In relationship	406	63.0	240	68.6	166	56.5		

*p ≤ .01

### Procedure

The current online questionnaire study was part of a larger study regarding adult pornography consumption and sexual health (Komlenac & Hochleitner, 2020, 2021). The study was conducted among medical students at an Austrian medical university. An e-mail invitation to participate in the current study was sent to all medical students at this medical university in March 2019. During this period, 3,201 students (55% women, 46% men) were enrolled at the medical university (Unidata, 2020). The e-mail invitation included information on the goal of the study, participation conditions, and a link to access the survey. The questionnaire was hosted on *SoSci: der onlineFragebogen* (http://soscisurvey.de/). One participation reminder was sent to the same students in April 2019. A last reminder was sent in May 2019. The online questionnaire study was accessible until the middle of June 2019. In total, *N* = 825 people accessed the online questionnaire, which resulted in an estimated response rate of 26%. Participation was voluntary, anonymous and not associated with any compensation. Participants could open the questionnaire only after giving informed consent. They were able to withdraw from participation and delete all their given answers at any time. Participants needed on average 13.5 min (SD = 4.8) to complete the questionnaire.

The medical university’s ethics committee exempted the current study from full ethics review. The study was conducted in accordance with the Declaration of Helsinki (World Medical Association, 2013) and the standards of the American Psychological Association (APA, 2002).

### Measures

#### Sociodemographic Variables

The first part of the questionnaire contained questions about sociodemographic variables. Participants were asked for their self-reported gender (woman, man, other), age, and nationality (Austrian, German, Italian, other). Additionally, relationship status (single, in relationship) and sexual orientation (heterosexual, gay-identified/lesbian-identified, bisexual, asexual, other) were assessed. For any participant who chose another as a response, an additional open text field allowed the response to be specified. Because of the gender-specific questions about sexual functioning in the current study, only participants who self-identified as a woman or a man were considered in the analysis. None of these participants reported having an asexual or other sexual orientation. Furthermore, the questionnaire contained questions about pornography consumption, perceived realism of pornography, partnered sexual activity, sexual flexibility when sexual problems occur, and sexual functioning.

#### Pornography Consumption

Before asking participants about their pornography consumption, the following definition was given: pornography is “material [e.g., pictures, films, videos or text] deemed sexual, given the context, that has the primary intention of sexually arousing the consumer, and is produced and distributed with the consent of all persons involved” (McDonald & Kirkman, 2019, p. 163). A time frame-related assessment of pornography consumption was used (Solano et al., 2020). In two items, participants were asked how often they consumed pornography. The first item assessed whether participants had consumed pornography during their lifetime (“Have you ever consumed pornography?”). Participants were asked to give one of the following responses: Never vs. Once, and I would never do it again vs. Yes, but not in the past year vs. Yes, in the past year (Solano et al., 2020). Participants who responded that they had consumed pornography in the past year were asked to specify how often during the last 2 months they had consumed pornography (Less than once a month vs. 1–3 times a month vs. 1–2 times a week vs. 3–6 times a week vs. 7 or more times a week). For the analyses, the two items concerning the frequency of pornography consumption were combined into one new variable. This new variable combined the responses Never and Once, and I would never do it again into one value. The other responses included Yes, but not in the past year vs. Less than once a month vs. 1–3 times a month vs. 1–2 times a week vs. 3–6 times a week vs. 7 or more times a week.

#### Perceived Realism of Pornography

Two self-constructed items assessed the extent to which participants perceived pornography as realistic. These items were “I think sexual acts depicted in pornographic material are realistic” and “Sexual acts in pornographic material are similar to my real sexual activities.” Similar items were used in previous studies (Peter & Valkenburg, 2006, 2010; Wright & Stulhofer, 2019). Participants were asked to indicate their agreement or disagreement with the statements about pornography’s realism on a five-point Likert scale (1 = *strongly disagree*, 5 = *strongly agree*). Mean scores for these two items were calculated. Higher scores indicated that participants perceived pornographic material as more realistic. This variable had an internal consistency equal to a Cronbach’s α of 0.79 (2 items) for female participants and 0.70 (2 items) for male participants.

#### Partnered Sexual Activity

To assess the frequency of partnered sexual activity, participants were asked “How often in the last 2 months have you had partnered sexual activity (penis-vaginal sex, oral sex, anal sex or other forms)?” A similar response scale regarding the frequency of pornography consumption was used for this question. Participants could choose from the following responses: Not at all vs. Less than once a month vs. 1–3 times a month vs. 1–2 times a week vs. 3–6 times a week vs. 7 or more times a week.

#### Sexual Flexibility When Sexual Problems Occur

The SexFlex Scale (Gauvin & Pukall, 2018) assessed participants’ sexual flexibility when sexual problems occurred. Participants were asked how easily they “adaptively change [their] thoughts and behaviors in response to a sexual problem” (Gauvin & Pukall, 2018, p. 385). This scale consists of six statements about being able to change one’s approach to one’s current sexual activity when sexual problems arise. One item of the scale was, “I immediately change my approach to sex if a certain approach doesn’t work”. Participants were asked to indicate on a four-point Likert scale (1 = *seldom or never*; 4 = *almost always*) how often they were able to change their approach as described in the statements. High scores indicated a high level of sexual flexibility. Originally, this scale was reported to have a reliability equal to a Cronbach’s α of 0.88–0.89 (Gauvin & Pukall, 2018). An exploratory factor analysis (EFA) that used principal component analysis (PCA) for factor extraction and the Kaiser criterion (eigenvalues > 1) to determine the number of retained factors revealed a one-factor solution for women and men. In women, this factor explained 65.0% of the variance. In men, one factor explained 58.2% of the variance. Factor loadings of the items ranged from 0.68 to 0.87. In the current study, the SexFlex Scale had a Cronbach’s α of 0.90 (6 items) for female participants and 0.85 (6 items) for male participants. The items of the scale were translated into German with the forth-and-back procedure (Brislin, 1970). One German native speaker and professional translator translated the items from English to German. Another English native speaker and professional translator translated the German version back to English without knowledge of the original English version. Finally, the two English versions were compared by the first author, and any differences in wording were discussed with the translators until consensus on the final wording was reached (Brislin, 1970).

#### Sexual Functioning

To assess female participants’ sexual functioning, the 6-item version of the Female Sexual Function Index (FSFI-6) in the German language was used (Isidori et al., 2010). The FSFI-6 is a shortened form of the widely used Female Sexual Function Index (FSFI) (Rosen et al., 2000). The FSFI-6 uses only one item to assess each domain of sexual functioning (i.e., sexual desire, arousal, lubrication, orgasm, sexual satisfaction and pain during vaginal penetration) during the past 4 weeks. Responses were given on a 6-point Likert scale (0 = no sexual activity; 1 = low functioning in certain domains, 5 = high functioning in certain domains). The total score for sexual functioning was calculated by adding all six responses, whereby high scores indicated high levels of sexual functioning. This version was reported to have a reliability equal to a Cronbach’s α of 0.79 (Isidori et al., 2010). The EFA with PCA determined a one-factor solution with the Kaiser criterion. That factor explained 50.8% of the variance. Factor loadings ranged from 0.50 to 0.85. In the current study, the FSFI-6 had a Cronbach’s α of 0.79 (6 items).

Male participants’ sexual functioning was assessed with the erectile functioning scale of the International Index of Erectile Function (IIEF) (Rosen et al., 1997). This scale assesses erectile functioning with six questions. Answers were given on a 6-point Likert scale (0 = no sexual activity; 1 = low erectile functioning, 5 = high erectile functioning). Originally, these questions referred to erectile functioning within the past 4 weeks. To be more in accordance with classification time frames of sexual dysfunctions, the questions in the current study assessed male participants’ erectile functioning during the last 6 months (American Psychiatric Association, 2013). The total score for erectile functioning was calculated by adding the score for all six responses, whereby high scores indicated a high level of erectile functioning. Originally, this scale was reported to have a reliability higher than a Cronbach’s α of 0.92 (Rosen et al., 1997). The German-language version was reported to have a Cronbach’s α of 0.94 (Wiltink et al., 2003). In the current study, the EFA with PCA and the Kaiser criterion indicated that two items (IIEF Item 1 and IIEF Item 15) did not load on the same factor as did the other items. After removing those two items, a one-factor solution explained 88.7% of the variance. Factor loadings ranged from 0.92 to 0.96. The modified scale of the IIEF had a Cronbach’s α of 0.96 (4 items).

### Statistical Analysis

Of the 825 persons who accessed the online questionnaire, 15 did not open the questionnaire because they did not give informed consent. Of those persons who opened the questionnaire, 39 were excluded from analysis because they either did not report their self-identified gender or did not identify as a woman or a man. Three participants did not report their age. Additionally, 124 participants had to be excluded from the analysis because they did not provide full responses regarding pornography consumption, perceived realism, the SexFlex Scale, the IIEF or the FSFI-6. In the end, there were *n* = 644 full responses for analysis.

Variables did not markedly violate the assumption of a normal distribution (skew: − 1.55–0.94; kurtosis: − 1.03–2.41) (Weston & Gore, 2006). Descriptive statistics concerning participants’ responses included the percentages and means of given responses. Gender differences were calculated with *t*-tests or with a chi-square test for categorical variables. Correlations were calculated to analyze associations between sociodemographic variables, pornography consumption, perceived realism, frequency of partnered sexual activity, sexual flexibility, and sexual functioning.

**Fig. 1:**
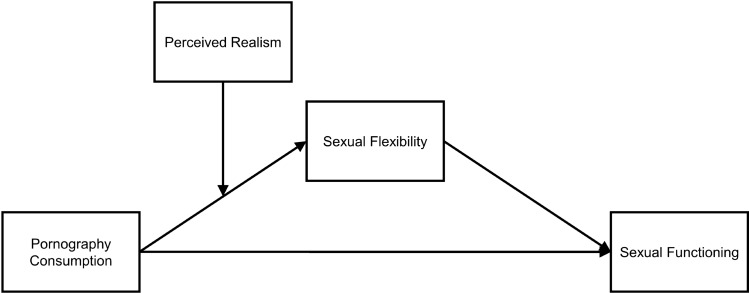
Manifest path model predicting sexual functioning

To analyze the main hypotheses of the study, manifest path analyses were conducted separately for female and male participants. A model was formulated to analyze associations between pornography consumption, perceived realism, sexual flexibility, and sexual functioning while controlling for sociodemographic variables and frequency of partnered sexual activity. This model is shown in Fig. 1. In the model, the direct and indirect associations between pornography consumption and sexual functioning were estimated. Sexual flexibility was entered as mediator. The interaction term of Pornography consumption x Perceived realism was entered as a predictor for sexual flexibility. Furthermore, it was analyzed whether indirect associations between pornography consumption and sexual functioning were moderated by perceived realism (Fig. 1). Age, frequency of partnered sexual activity, and sexual orientation were entered as covariables. For the statistical analyses, the Statistical Package for the Social Sciences (SPSS) for Windows, version 25.0 (IBM Corp., Armonk, NY, USA) was used. The manifest path analyses were performed with the PROCESS macro for SPSS. We estimated bootstrap bias-corrected 95% confidence intervals (bootstrap sample was *n* = 5000) for all path coefficients (Hayes, 2018; www.processmacro.org). Significant results were indicated when *p* ≤ .05 or when the 95% confidence intervals did not include zero.

## Results

### Descriptive Statistics

More female (19.1%) than male (1%) participants reported that they had never consumed pornography. One male participant (0.3%) compared to 7.1% of female participants had consumed pornography in the past but claimed that they would not do so again. More female (17.4%) than male (2.7%) participants reported that their last pornography consumption dated more than 1 year in the past. Male participants (95.9%) were more likely to have consumed pornography during the last 12 months than were female participants (56.3%; *χ*^2^(3) = 132.6, *p* < .001). Among those participants who had consumed pornography during the last 12 months, male participants (*M* = 4.1, SD = 1.2) consumed pornography on average more frequently (1–2 times a week) than did female participants (1–3 times a month; *M* = 2.5, SD = 1.2; *t*(534.7) = 15.2, *p* < .001, *r* = .51). On average, women and men disagreed that pornography was realistic (Table 2).

**Table 2:** Descriptive statistics

Variable	Full sample		Women		Men		*t*(642)	*p*	*r*
	*M*	SD	*M*	SD	*M*	SD			
Pornography consumption	2.9	1.8	1.9	1.5	4.1	1.3	19.4	< .001	.61
Perceived realism	2.0	0.8	2.0	0.9	2.1	0.8	0.8	.448	
Partnered sexual activity	3.4	1.4	3.6	1.2	3.1	1.5	− 4.8	< .001	.19
Sexual flexibility	2.7	0.7	2.7	0.7	2.6	0.7	− 3.0	.003	.19
Sexual functioning^a^			22.8	5.2	16.1	6.7			

^a^Women’s sexual functioning was assessed with the Female Sexual Function Index and men’s sexual functioning was assessed with the erectile functioning scale of the International Index of Erectile Function. The two scales contain different items. This is why gender differences in this variable were not calculated

Approximately 9.7% of the female participants and 23.8% of the male participants reported that they had not had any partnered sexual activity in the previous 2 months. Of those participants who had partnered sexual activity, men reported having had such activity on average 1–3 times a month, whereas women reported having had such activity on average 1–2 times a week (Table 2).

Female participants reported, on average, more sexual flexibility when sexual problems occurred than did male participants (Table 2). The average FSFI-6 score indicated that most female participants reported a satisfying level of sexual functioning (Table 2, Isidori et al., 2010). The average IIEF score showed that most of the participants reported having no erectile problems (Table 2, Rosen et al., 1997).

### Correlation Analysis

The correlations between pornography consumption, perceived realism, sexual flexibility, sexual functioning, and sociodemographic variables are shown in Table 3. In the female sample, frequent pornography consumption was correlated with greater perceived realism of pornography, greater sexual flexibility and greater sexual functioning. Furthermore, nonheterosexual sexual orientation was associated with more frequent pornography consumption. Women who were currently in a relationship reported, on average, a lower frequency of pornography consumption, a greater frequency of partnered sexual activity, and greater sexual functioning than did single women. Frequent partnered sexual activity was linked to greater perceived realism, greater sexual functioning and greater sexual flexibility. Greater sexual flexibility was additionally associated with greater sexual functioning in women. Finally, greater perceived realism was associated with greater sexual flexibility and greater sexual functioning (Table 3).

**Table 3:** Intercorrelations for study variables disaggregated by gender

	1	2	3	4	5	6	7	8	9
1. Age	–	− .01	− .02	.14*	− .02	− .01	.03	− .07	.11*
2. Nationality	− .03	–	.03	− .10	.06	− .04	.00	.00	.02
3. Sexual orientation	− .01	.03	–	.05	.08	.28**	.14*	.16**	.04
4. Relationship	.19**	.09	− .08	–	.54**	− .12*	.02	− .03	.29**
5. Partnered sexual activity	.14*	.03	.04	.62**	–	.00	.14**	.20**	.50**
6. Pornography consumption	.02	− .02	.11	− .21**	− .14*	–	.39**	.23**	.16**
7. Perceived realism	.11	− .07	.01	.09	.26**	.03	–	.14**	.15**
8. Sexual flexibility	− .05	− .06	.10	.09	.19**	.11	.08	–	.27**
9. Sexual functioning	.10	.02	− .03	.39**	.62**	.00	.21**	.15*	–

The results for the female sample (*n* = 350) are shown above the diagonal. The results for the male sample (*n* = 294) are shown below the diagonal

*p ≤ .050, **p ≤ .01

In the male sample, frequent pornography consumption was correlated with a reduced frequency of partnered sexual activity and with being single. Men who were currently in a relationship reported, on average, greater sexual flexibility and greater perceived realism of pornography than did single men. Men’s greater sexual functioning was associated with frequent partnered sexual activity, greater perceived realism, greater sexual flexibility, and being in a relationship (Table 3).

### Direct and Indirect Associations Between Pornography Consumption and Sexual Functioning

All path coefficients of the manifest path analysis for the female sample are shown in Table 4. The model explains 10% of the variance in sexual flexibility (*F*(6, 343) = 6.6, *p* < .001). Frequent partnered sexual activity and frequent pornography consumption were linked to greater sexual flexibility. Neither other main effects nor the interaction term of Pornography consumption x Perceived realism was significantly associated with sexual flexibility.

**Table 4:** All model path coefficients in the female sample

Variable	*B*	*SE B*	95% CI for *β*		*R*^2^
			LL	UL	
*Outcome variable: Sexual flexibility*					.10*
Age	− 0.01	0.01	− 0.03	0.01	
Sexual orientation	0.96	0.06	− 0.02	0.21	
Partnered sexual activity	0.11	0.03	0.05	0.17^a^	
Pornography consumption	0.09	0.02	0.04	0.15^a^	
Perceived realism	0.03	0.05	− 0.06	0.13	
Pornography consumption × Perceived realism flexibility	− 0.01	0.02	− 0.06	0.03	
*Outcome variable: Sexual functioning*					.31*
Age	0.18	0.06	0.06	0.30^a^	
Sexual orientation	− 0.47	0.37	− 1.20	0.27	
Partnered sexual activity	1.98	0.19	1.60	2.35^a^	
Pornography consumption	0.48	0.16	0.17	0.80^a^	
Sexual flexibility	1.15	0.34	0.48	1.83^a^	
Indirect associations with sexual functioning (low perceived realism)					
Pornography consumption $\to$ Sexual flexibility $\to$ Sexual functioning	0.12	0.06	0.02	0.26^a^	
Indirect associations with sexual functioning (high perceived realism)					
Pornography consumption $\to$ Sexual flexibility $\to$ Sexual functioning	0.10	0.05	0.01	0.19^a^	

*CI* confidence interval

*p ≤ .001

^a^Significant effect indicated by 95% confidence interval not containing both positive and negative values

The model explains 31% of the variance in women’s sexual functioning (*F*(5, 344) = 31.3, *p* < .001). Age, frequency of partnered sexual activity, sexual flexibility and pornography consumption were directly linked to female sexual functioning. Additionally, frequent pornography consumption was indirectly linked to women’s greater sexual functioning through greater sexual flexibility, irrespective of perceived realism (Table 4).

All path coefficients of the manifest path analysis for the male sample are shown in Table 5. The model explains 7% of the variance in male participants’ sexual flexibility (*F*(6, 287) = 3.4, *p* = .003). Similar to the female model, in the male path model, frequent partnered sexual activity and frequent pornography consumption were linked to greater sexual flexibility. Neither other main effects nor the interaction term of Pornography consumption x Perceived realism was significantly associated with sexual flexibility.

**Table 5:** All model path coefficients in the male sample

Variable	*B*	SE *B*	95% CI for *β*		*R*^2^
			LL	UL	
*Outcome variable: Sexual flexibility*					.07*
Age	− 0.02	0.01	− 0.04	0.01	
Sexual orientation	0.10	0.08	− 0.06	0.26	
Partnered sexual activity	0.10	0.03	0.04	0.15^a^	
Pornography consumption	0.07	0.03	0.01	0.13^a^	
Perceived realism	0.03	0.05	− 0.08	0.14	
Pornography consumption × Perceived realism flexibility	− 0.02	0.04	− 0.10	0.05	
*Outcome variable: Sexual functioning*					.39**
Age	0.02	0.08	− 0.15	0.18	
Sexual orientation	− 0.87	0.62	− 2.10	0.35	
Partnered sexual activity	2.83	0.22	2.40	3.26^a^	
Pornography consumption	0.46	0.25	− 0.03	0.95	
Sexual flexibility	0.26	0.45	− 0.63	1.16	
Indirect associations with sexual functioning (low perceived realism)					
Pornography consumption $\to$ Sexual flexibility $\to$ Sexual functioning	0.02	0.05	− 0.05	0.15	
Indirect associations with sexual functioning (high perceived realism)					
Pornography consumption $\to$ Sexual flexibility $\to$ Sexual functioning	0.01	0.03	− 0.05	0.09	

*CI* confidence interval

*p ≤ .01 **p ≤ .001

^a^Significant effect indicated by 95% confidence interval not containing both positive and negative values

The model explains 39% of the variance in men’s sexual functioning (*F*(5, 388) = 36.9, *p* < .001). However, the only variable that was significantly linked to men’s sexual functioning was partnered sexual activity. No other direct or indirect associations with men’s sexual functioning were determined (Table 5).

## Discussion

The current study found a direct association between frequent pornography consumption and greater sexual functioning in women but not in men (H1). Additionally, an indirect link between frequent pornography consumption and greater sexual functioning through the mediator of sexual flexibility was found in women (H2). The perceived realism of pornographic material did not moderate the found associations (H3).

### Frequent Pornography Consumption and Greater Sexual Functioning in Women

Our study supports previous findings that revealed no associations between men’s pornography consumption and sexual functioning (Landripet & Štulhofer, 2015; Prause & Pfaus, 2015). Moreover, our study adds to the literature by providing findings on such associations for women. In contrast to other studies that have analyzed this link (Berger et al., 2019; Wright et al., 2021), we found an association between women’s frequent pornography consumption and greater sexual functioning (Bőthe et al., 2021).

The majority of pornographic content depicts two actors who engage in genital stimulation, oral stimulation, or vaginal intercourse (Gorman et al., 2010; Vannier et al., 2014). Such pornographic material may help women expand their sexual scripts, learn new rewarding sexual behaviors, and thereby increase their sexual flexibility. For example, learning about oral-genital activity has been most frequently cited in this regard (Weinberg et al., 2010). This finding is supported by additional findings showing that women who consume pornography more frequently have oral sexual activity than do women who do not consume pornography (Brown & L'Engle, 2009).

The current finding of associations between frequent pornography consumption and greater sexual functioning is further supported by studies that report consumers’ self-perceived effects of their pornography consumption. Such studies report that people self-perceive positive effects of pornography consumption on their sexuality rather than negative consequences (Daneback et al., 2009; Hald & Malamuth, 2008; Weinberg et al., 2010).

The current study’s findings, as well as the findings of past studies, may encourage some clinical practitioners to use pornography in psychosexual therapy to instruct or show clients new or alternative sexual behaviors (Brewster & Wylie, 2008). Such material may, for example, include portrayals of adult solo and mutual masturbation and oral, vaginal, and anal sexual activity. Such material may encourage clients to explore alternative sexual activities when problems occur during preferred sexual behaviors. Furthermore, such material may help with the understanding and acceptance of certain sexual behaviors (Watson & Smith, 2012). However, one must also bear ethical implications in mind when using pornography in psychosexual therapy. Some clients might find the use of pornographic material challenging and distressing because of their attitudes or past experiences (Rhoades, 2007). Therefore, clinicians must evaluate a client’s readiness to view pornographic material. Additionally, some other sexual health concerns, such as relationship problems, may contraindicate the use of pornographic material (Miller et al., 2019; Wright et al., 2017).

### Alternative Explanations

Our hypothesis that the link between pornography consumption and sexual functioning is stronger for people who perceive pornography as realistic than for people who do not perceive such content to be realistic was not supported by our findings. As is the main problem with most cross-sectional studies, the current cross-sectional study does not permit any conclusions about the directionality or causality of the found associations. The associations found between pornography consumption and sexual flexibility could mean that women who are already open and flexible in their approach to sexuality are more likely to consume pornography than are women who are limited in regard to their sexual flexibility. In such a case, one’s perceived realism of pornography is unlikely to influence the association.

The Antecedents-Context-Effects Model (Campbell & Kohut, 2017) exemplifies the problem that most of the studies about pornography do not consider the factors or traits that make a person more likely to consume pornography. Thus, many studies do not consider the so-called antecedents of pornography consumption. In some cases, those antecedents and not pornography consumption may be a better explanation for the found links between pornography consumption and components of sexual health (Campbell & Kohut, 2017). This also applies to our study and the hypothesized associations between sexual flexibility, sexual functioning and pornography consumption. Future studies of the links between pornography consumption and sexual health should consider potential factors that may explain pornography consumption and assumed effects of pornography consumption. Additionally, future longitudinal studies or experimental studies are needed to shed light on the directionality of the found associations.

Even though pornography consumption was seen to be indirectly associated with sexual functioning through sexual flexibility, sexual flexibility could not fully explain the association between pornography consumption and sexual functioning. The considerable direct effect between pornography consumption and sexual functioning indicates that further studies need to include additional mediators to explain the found associations. For example, women who consume pornography may be more likely to know their own sexual interests and desires and in turn be willing and able to communicate their preferences during partnered sexual activity (Weinberg et al., 2010). The ability to communicate sexual preferences has been reported to be associated with greater sexual satisfaction in women (Blunt-Vinti et al., 2019; Herbenick et al., 2019).

### Gender Differences

The current study replicated the previously known finding that men consume pornography more frequently than women (Landripet & Štulhofer, 2015; Miller et al., 2019; Sun et al., 2016). Additional gender differences became evident, as hypothesized associations between pornography consumption, sexual flexibility, and sexual functioning were supported only in women but not in men.

Notably, an association was observed between frequent pornography consumption and greater sexual flexibility in men. However, sexual flexibility, in turn, was not linked to sexual functioning. One explanation for the different findings in women and men may be explained by the methods used to assess sexual functioning in women and men. For women, we used the FSFI-6, which includes many domains of sexual functioning. The erectile functioning scale of the IIEF, in contrast, contains questions only about erectile functioning. Thus, associations between men’s frequency of pornography consumption and other components of sexual functioning may have been missed because this scale was used. There is evidence that pornography consumption may be positively associated with men’s sexual desire (Prause & Pfaus, 2015). Future studies should use more sophisticated questionnaires that assess each component of sexual functioning with multiple items. Furthermore, future studies may also include questions about the distress a sexual problem causes because sexual problems that cause considerable distress may be clinically relevant. Prevalence rates and estimates of sexual problems change significantly when distress is considered (Hendrickx et al., 2016; Komlenac et al., 2019; Mitchell et al., 2016).

The purpose of pornography consumption may also differ between women and men. Compared to women, men are more likely to use pornography to achieve sexual arousal during solitary sexual activity. Women are more likely than men to report using pornography together with a partner to enhance sexual stimulation during partnered sexual activity (Albright, 2008; Bridges & Morokoff, 2011; Solano et al., 2020). Additionally, gender differences have been reported in regard to preferences for specific pornographic content (Hald & Štulhofer, 2016), for pornographic material (e.g., pictures, films, videos or text) accessed, or for the motivation behind or the purpose of pornography consumption (Solano et al., 2020). All these factors may influence the effects of pornography consumption (Wright, 2011) and may explain the gender differences in the found associations between pornography consumption and components of sexual health, including sexual functioning. Therefore, we agree with recommendations that future studies should extend their measures of pornography consumption to include questions on frequency, content, medium, and motivation (Hald & Štulhofer, 2016; Solano et al., 2020).

### Limitations

The current study is not without its limitations. First, the study is based on participants’ self-reports. This approach entails known problems. For instance, participants may not correctly remember all occasions of their sexual activity or pornography consumption. Additionally, participants may have felt that it is socially desirable to withhold or reveal certain information (Choi & Pak, 2005).

Second, the found associations are only small or moderate (Cohen, 1988). Therefore, the found associations between sexual functioning, sexual flexibility, and pornography consumption should be interpreted with caution.

Third, we modified the questions on the IIEF (Rosen et al., 1997) from asking about sexual functioning in the previous 4 weeks to asking about sexual functioning over the last 6 months to be more in accordance with classification time frames for sexual dysfunctions (American Psychiatric Association, 2013). However, a technical error caused us to not apply the same changes to the FSFI-6 (Isidori et al., 2010). Future studies should include a 6-month time frame for women and men.

Last, even though the study used a relatively large sample, this sample has limitations. The current study’s results are based on a convenience sample of university students. Many other studies of pornography consumption have used such samples (Short et al., 2012). However, such a sample may significantly differ from other populations (Henrich et al., 2010). In general, studies with university students as participants find associations with larger effect sizes than those of studies with more general samples. Additionally, it has been shown that the directionality of an association may be in the opposite direction in studies with university students and in studies with nonstudent samples. This is why conclusions based on studies with only university students as participants may differ from studies that base their findings on a less homogeneous and more general sample (Peterson, 2001). Another limitation of the sample is that sexual minority groups remained relatively underrepresented. Found associations between sexual orientation and pornography consumption indicate that acceptance and habits of pornography consumption may differ between people of different sexual orientations. These limitations indicate the need for future studies with more diverse samples to replicate and extend current findings.

### Conclusion

Although an association between pornography consumption and sexual functioning has often been proposed (Ley et al., 2014), to date, only a few studies have examined this association. The current findings revealed direct and indirect associations between frequent pornography consumption and greater sexual functioning in women but not in men. The indirect association between pornography consumption and sexual functioning in women was partially mediated by sexual flexibility. Thus, women may expand their sexual scripts and learn new sexual behaviors from pornography consumption. Having sexual scripts that are alternative to one’s preferred sexual activity may help people switch to alternative rewarding sexual behavior when sexual problems arise.

However, many questions also remained unanswered in the current study and warrant further investigation. First, a significant proportion of variance in sexual functioning was directly explained by pornography consumption. Future studies are needed to explain the mechanism between the found associations. Additionally, gender differences need to be better addressed in future studies. The current study replicated the previously known finding that men consume pornography more frequently than do women (Landripet & Štulhofer, 2015; Miller et al., 2019; Sun et al., 2016). The current study also replicated findings that reveal no associations between men’s pornography consumption and sexual problems (Landripet & Štulhofer, 2015; Prause & Pfaus, 2015). However, future studies with more precise measures of pornography consumption that include measures of frequency, content, medium, and motivation are needed to explain gender differences (Hald & Štulhofer, 2016; Solano et al., 2020).
